# Low Fluoride Regulates Macrophage Polarization Through Mitochondrial Autophagy Mediated by PINK1/Parkin Axis

**DOI:** 10.3390/biom15050647

**Published:** 2025-04-30

**Authors:** Fengyu Xie, Jing Zhou, Bingshu Liu, Lijun Zhao, Cunqi Lv, Qiong Zhang, Lin Yuan, Dianjun Sun, Wei Wei

**Affiliations:** 1 Center for Endemic Disease Control, Chinese Center for Disease Control and Prevention, Harbin Medical University, Harbin 150081, China; 2022020100@hrbmu.edu.cn (F.X.); zhoujing@ems.hrbmu.edu.cn (J.Z.);; 2 Key Lab of Etiology and Epidemiology, Education Bureau of Heilongjiang Province, Ministry of Health of PR, Harbin Medical University, Harbin 150081, China

**Keywords:** low fluoride, macrophage polarization, spleen inflammation, mitophagy, PINK1/Parkin pathway

## Abstract

Fluoride exposure has been shown to affect immune cell subsets and immune function, but its impact on macrophage polarization remains unclear. This study investigates the effects of low fluoride exposure on macrophage polarization and its underlying mechanisms through epidemiological surveys, animal experiments, and in vitro cell experiments. In the population-based epidemiological survey, we used mass cytometry to assess the impact of low fluoride exposure (0.570–2.027 mg/L) in the environment on human immune cell populations following the current water improvement and fluoride reduction measures. A rat fluorosis model was established by treating rats with sodium fluoride (NaF) in drinking water at concentrations of 0 mg/L, 5 mg/L, 10 mg/L, 25 mg/L, and 50 mg/L for 90 days., and morphological changes were assessed by hematoxylin–eosin (H&E) staining and transmission electron microscopy in the spleen of rats. Flow cytometry was used to analyze the proportion of macrophage subtypes in the spleen, while Western blot and immunofluorescence were performed to detect the expression of mitochondrial autophagy-related proteins. An M1 macrophage model was constructed in vitro by inducing THP-1 cells, and the effects of fluoride on macrophage-related cell markers and cytokines were assessed using flow cytometry and ELISA, respectively, following intervention with an autophagy inhibitor. Mitochondrial membrane potential and mitochondrial–lysosomal colocalization are analyzed through flow cytometry and confocal microscopy. The study aims to investigate the role of mitophagy in sodium fluoride-induced macrophage polarization. Epidemiological investigations revealed that low fluoride increases the proportion of blood monocytes, as well as the expression levels of CD68 (a macrophage surface marker), CD86 (an M1 macrophage marker), and the inflammatory cytokine IFN-γ in peripheral blood mononuclear cells (PBMCs). In the rats of NaF-treated groups, splenic tissues exhibited inflammatory infiltration, mitochondrial swelling, and increased autophagosome formation. Moreover, low fluoride activated the PINK1/Parkin-mediated mitophagy pathway, promoting an increase in the M2/M1 macrophage ratio. In vitro experiments further confirmed that autophagy inhibitors reversed the NaF-induced increase in the M2/M1 macrophage ratio. This study demonstrates that low fluoride induces inflammatory responses in the body and drives M1 macrophage polarization toward M2 macrophages via mitophagy. These findings highlight the potential immunological risks associated with low fluoride and provide mechanistic insights into the interplay among fluoride, mitophagy, and macrophage polarization.

## 1. Introduction

Fluorine, a highly reactive non-metallic element, is widely distributed in air, water, and soil in various compound forms. However, its distribution in the Earth’s crust is uneven [1], leading to the presence of fluorosis-endemic areas in countries such as China [2], Iran [3,4], Pakistan [5], and India [6]. Environmental fluoride exposure poses a significant global public health challenge.

Fluoride can damage multiple organs and systems [7], including the ovaries [8], liver [9], kidneys [10], brain [11], skeletal system [12], reproductive system [13], and immune system [14]. The immune system is a critical line of defense against external pathogens, and its dysfunction increases the risk of infections and various diseases [15]. Macrophages, key players in inflammation, immune responses, and tissue homeostasis, exhibit high plasticity. Depending on the microenvironment, macrophages can differentiate into two major activation states: classically activated (M1) and alternatively activated (M2) macrophages [16,17,18,19]. M1 macrophages possess strong pro-inflammatory properties and play roles in antimicrobial and antitumor activities. In contrast, M2 macrophages are involved in phagocytosis, parasite clearance, inflammation suppression, tissue repair, and tumor progression [20,21].

During tissue damage or infection, monocytes increase in number [22] and migrate to the site of injury, differentiating into different macrophage subtypes based on the microenvironment [23]. In the early stages of inflammation, macrophages adopt a pro-inflammatory phenotype to activate antimicrobial defense mechanisms and promote inflammation [24]. However, if the pro-inflammatory response is not controlled, it becomes pathogenic, contributing to disease progression. To mitigate the potential harm of pro-inflammatory responses, these macrophages undergo apoptosis or shift to an anti-inflammatory phenotype, promoting inflammation resolution and tissue repair [25]. High fluoride levels can alter macrophage polarization. Studies have shown that exposure to high fluoride (1 mol/L) induces M1 macrophage polarization in mouse periodontal tissue, exacerbating periodontitis [26]. Similarly, high fluoride (10 mmol/L) in fracture tissue increases M1 polarization while reducing M2 polarization, delaying bone healing in rats [27].

Currently, there is no research on the effects of low fluoride levels on macrophage polarization. However, in vitro studies suggest that low fluoride reduces ATP production and increases reactive oxygen species (ROS) generation in macrophages [28]. Even at low concentrations, fluoride can influence the activity and expression of oxidases, participating in the initiation and progression of inflammation [29].

Mitochondria, the primary source of cellular energy, play a crucial role in regulating eukaryotic cellular functions. Under normal physiological conditions, PTEN-induced putative kinase 1 (PINK1) is transported to the mitochondrial inner membrane and degraded. In cases of mitochondrial damage, mitochondrial membrane potential (MMP) decreases, preventing PINK1 transport and causing its accumulation on the outer membrane of damaged mitochondria, where it recruits Parkin [30]. Parkin initiates ubiquitination and recruits receptor proteins such as p62/SQSTM1 (p62) protein, which bind to microtubule-associated protein 1 light chain 3 (LC3) on the phagophore membrane. The phagophore subsequently matures into an autophagosome, which fuses with lysosomes to degrade damaged mitochondria [31]. Studies have reported that fluoride induces mitochondrial damage and mitophagy in stromal cells of the testis [32], and hepatocytes [33]. Mitophagy has been shown to regulate macrophage polarization, promoting M2 polarization while inhibiting mitophagy shifts macrophages toward the M1 phenotype [34].

Governments worldwide have implemented water defluoridation programs with significant success. For instance, the U.S. Public Health Service (USPHS) recommends that community water systems maintain fluoride concentrations below 700 µg/L to maximize dental caries prevention while minimizing the risk of dental fluorosis [35]. China and India have set a safe fluoride limit of 1 mg/L for drinking water [36,37], while Iran has adopted the World Health Organization (WHO) standard of 1.5 mg/L [38]. Asian countries are also developing novel nanomaterials for fluoride removal [36], employing methods such as adsorption, electrocoagulation, nanofiltration, and ion exchange to reduce fluoride levels in groundwater [6]. These measures have significantly reduced the number of high-fluoride exposure areas, lowering the prevalence of dental and skeletal fluorosis [39,40,41]. Given this progress, research on the effects of low fluoride exposure on the human body has become increasingly relevant. This study employs in vivo and in vitro experiments to evaluate the effects of low fluoride on macrophage polarization and explores the underlying mechanisms, providing further scientific evidence on the impact of fluoride on the immune system.

## 2. Materials and Methods

### 2.1. Materials, Reagents and Cell Culture

#### 2.1.1. Material Reagents

Chloroquine diphosphate salt (CQ) was purchased from Sigma-Aldrich (St. Louis, MO, USA). Phorbol 12-myristate 13-acetate (PMA) was obtained from Aladdin (Shanghai, China). JC-1 Mitochondrial Membrane Potential Assay Kit was provided by Solarbio (Beijing, China). Mitochondrial Green Fluorescent Probe, Lysosomal Red Fluorescent Probe, and BCA Protein Assay Kit were purchased from Beyotime (Shanghai, China). Anti-p62 (#8025/#23214) was obtained from Cell Signaling Technology (Danvers, MA, USA). Anti-LC3B (#ab192890) was purchased from Abcam (Cambridge, UK). Anti-PINK1 (23274-1-AP), anti-Parkin (14060-1-AP), and anti-GAPDH (60004-1-IG) were purchased from Proteintech (Wuhan, China). Anti-β-Actin (#AF7018) was obtained from Affinity Biosciences (Jiangsu, China). Fc Blocker (550270) was purchased from BD Biosciences (San Jose, CA, USA). Flow cytometry antibodies were obtained from BioLegend (San Diego, CA, USA): PerCP anti-rat CD45 (202220), FITC anti-rat CD86 (200305), PE anti-rat CD68 (201004), PerCP anti-human CD86 (374216), APC anti-human CD206 (321110), and PE anti-human CD68 (333808). APC anti-rat CD206 (sc-58986) was purchased from Santa Cruz Biotechnology (Dallas, TX, USA). ELISA kits for IL-1β, IL-10, TGF-β, and TNF-α were purchased from Enzyme-linked Biotechnology (Shenzhen, China).

#### 2.1.2. Cell Culture and Induction

The human acute monocytic leukemia cell line THP-1, a model cell line for in vitro monocyte-to-macrophage differentiation [17], was obtained from the Cell Bank of the Chinese Academy of Sciences (Shanghai, China). According to ATCC protocols, the cells were cultured in RPMI-1640 medium supplemented with 10% fetal bovine serum (FBS), 1% penicillin–streptomycin, and 0.1% β-mercaptoethanol. The cells were maintained at 37 °C in a humidified atmosphere containing 5% CO_2_. M0 macrophages were generated by treating THP-1 monocytes with PMA (20 ng/mL) for 24 h. To obtain M1 macrophages, THP-1 cells were first treated with 20 ng/mL PMA for 24 h, followed by treatment with 20 ng/mL IFN-γ and 20 ng/mL LPS for an additional 48 h.

### 2.2. Study Location and Population Information

A cross-sectional survey was conducted in July 2023 in five villages with varying levels of water fluoride exposure in Zhaodong City, Heilongjiang Province, China (Wujian Village, Hongqing Village, Wujing Village, Pingfang Village, and Hongguang Village). All participants signed informed consent forms and completed written information sheets for the questionnaire survey. Data were collected using a self-designed questionnaire administered by specially trained investigators. A total of 249 participants were included in the study. The inclusion criteria were as follows: 1. age ≥ 18 years; 2. clear consciousness and the ability to complete the questionnaire independently; 3. good health status with no history of severe chronic diseases or immune-related disorders; 4. signed informed consent and agreed to participate in the study and provide relevant samples. The exclusion criteria were as follows: 1. individuals with immune system diseases (e.g., AIDS and autoimmune diseases); 2. individuals currently using or with a history of long-term use of medications affecting the immune system (e.g., anti-inflammatory drugs). For each participant, drinking water, blood, and urine samples were collected. Urinary fluoride concentrations were categorized into three groups based on tertiles: Q1: ≤P33.3 (0.898 mg/L), Q2: (P33.3–P66.7] (0.898–1.432 mg/L], Q3: >P66.7 (1.432 mg/L).

### 2.3. Mass Cytometry for the Detection of Monocytes and Related Markers in Blood

Six participants were randomly selected from each of the three groups. Fasting venous blood samples (5 mL) were collected using EDTA anticoagulant tubes. Peripheral blood mononuclear cells (PBMCs) were isolated using Ficoll-Paque PREMIUM 1.073 (Cytiva, GE Healthcare, Chicago, IL, USA) following the manufacturer’s instructions. After isolation, the PBMCs were washed and resuspended in PBS. The cells were stained with a panel of 41 antibodies conjugated to heavy metal isotopes. After staining, the cells were analyzed using a mass cytometer (Helios, Fluidigm, South San Francisco, CA, USA). For each sample, 1 × 10^5^ cells were acquired and analyzed. The identification of monocyte subsets is based on the following markers: monocytes(CD11b⁺CD14⁺), classical monocytes(CD14^+^CD16), and non-classical monocytes(CD14^lo^CD16⁺).

### 2.4. Establishment of a Rat Fluorosis Model

Fifty healthy 3-week-old male Wistar rats were purchased from Beijing Vital River Laboratory Animal Technology Co., Ltd. (Beijing, China) (License No.: SCXK (Jing) 2021-0006). All the rats were housed in an SPF-grade animal facility under controlled temperature and humidity conditions, with a 12 h light/dark cycle. The rats had ad libitum access to water and chow. After one week of acclimatization, the rats were exposed to varying concentrations of NaF in drinking water based on Zhou et al.’s definition of low fluoride levels [7]. Low fluoride exposure was defined as NaF concentrations not exceeding 20.9 mg/L. The rats were randomly divided into five groups: control group (0 mg/L NaF), low fluoride groups (5 mg/L and 10 mg/L NaF), moderate fluoride group (25 mg/L NaF), high fluoride group (50 mg/L NaF). During the 12-week experimental period, the rats’ water consumption and body weight were recorded weekly. At the end of the study, the rats were euthanized under isoflurane anesthesia, and samples were collected, including photographs of teeth, blood, urine, and spleen tissues. The spleens were immediately dissected and weighed. All the animal experiments in this study complied with the Regulations for the Administration of Affairs Concerning Experimental Animals of the People’s Republic of China.

### 2.5. Quantitative Determination of Fluoride Content in Serum, Urine and Water

According to the National Health Industry Standards of the People’s Republic of China (WS/T212-2001) [42], “Determination of Fluoride in Serum” and (WS/T89-2015) [43] “Determination of Fluoride in Urine”, fluoride ion concentration in drinking water, urine, and blood (from both humans and rats) was detected using the fluoride ion-selective electrode method. After sample collection, drinking water samples can be directly tested without any pretreatment, while urine and blood samples require centrifugation to separate the supernatant and serum for testing. The fluoride ion concentration was determined using a calibration standard curve, which covers the range of 0.1–10 mg/L and shows a good linear relationship (R^2^ > 0.999).

### 2.6. Diagnostic Criteria for Dental Fluorosis

The dental fluorosis condition of each rat was evaluated and scored according to the modified Dean index [44]. In this study, the severity of dental fluorosis in the rats was classified into four levels: (1) normal—teeth are yellow-orange in color with a glossy surface; (2) mild—the tooth surface has opaque white streaks; (3) moderate—the area of white horizontal streaks expands, forming cloudy regions; (4) severe—the entire tooth surface appears opaque and chalky white, with some teeth showing damage.

### 2.7. Preparation of Spleen Cell Suspension

The spleen, excised from the body, was washed with PBS and then a quarter of the spleen was placed into a culture dish containing RPMI 1640 supplemented with 1% penicillin–streptomycin solution. The spleen was gently minced using sterile scissors and ground with a sterile pestle. The resulting spleen cell suspension was transferred to a 24-well plate, and collagenase VI was added. The plate was then placed in a 37 °C cell incubator for tissue digestion for 1 h. After filtration through a 200-mesh filter, the suspension was centrifuged, and the supernatant was discarded. The pellet was resuspended in an appropriate volume of red blood cell lysis buffer and incubated at room temperature for a few minutes. The cells were then centrifuged, the pellet collected, and resuspended in PBS for subsequent experiments.

### 2.8. H&E Histological Staining

The spleen was fixed in 4% paraformaldehyde for 48 h prior to histological analysis. The samples were dehydrated, embedded in paraffin, and sectioned into 4 μm thick slices. The sections were stained with hematoxylin and eosin, followed by dehydration. The stained sections were then mounted with resin and imaged using an inverted optical microscope.

### 2.9. Ultrastructural Observation

Macrophages, after centrifugation to form cell aggregates, were processed together with the spleen tissue specimens cut into approximately 1 mm^3^ blocks. The samples were first fixed in 2.5% glutaraldehyde, rinsed with 0.1% phosphate-buffered saline, and further fixed in 1% osmium tetroxide. The samples were dehydrated in acetone and then infiltrated with a 1:1 mixture of pure acetone and pure resin. Subsequently, the samples were embedded in pure resin overnight at 37 °C. After embedding, the tissue was sectioned into ultrathin sections of 50–70 nm using an ultramicrotome, and double-stained with uranyl acetate and lead citrate. The ultrastructure of the tissue and cells was observed using a transmission electron microscope (H-600; Hitachi High-Technologies Corporation, Tokyo, Japan), and images were captured.

### 2.10. Immunofluorescence Staining

Deparaffinized and dehydrated spleen tissue sections were incubated with primary antibodies against LC3 and P62 at 4 °C for 12 h, followed by incubation with secondary antibodies at 37 °C for 1 h. After washing, the sections were stained with DAPI. The fluorescence signals were observed using a confocal microscope (LSM 800, Zeiss, Oberkochen, Germany).

### 2.11. Flow Cytometry Analysis of Macrophage Phenotype

Spleen cells from the rats were resuspended in PBS (BL302A, Biosharp, Hefei, China). Fc block was added to the cell suspension, and the cells were incubated at 4 °C for 10 min. Then, PerCP anti-rat CD45 and FITC anti-rat CD86 antibodies were added, and the cells were incubated at 4 °C for 30 min. After washing with PBS, the cells were permeabilized and incubated with APC anti-rat CD206 and PE anti-rat CD68 antibodies at 4 °C for 30 min. For the M1 macrophages derived from THP-1 cells, Fc block was added and incubated at 4 °C for 10 min. PerCP anti-human CD86 was then added, and the cells were incubated at 4 °C for 30 min. After permeabilization, APC anti-human CD206 and PE anti-human CD68 antibodies were added and incubated at 4 °C for 30 min. All the samples were analyzed for fluorescence intensity using a flow cytometer (FACS Melody, BD Biosciences, San Jose, CA, USA) and analyzed with FlowJo 10.8.1 software. The spleen cell samples were collected at 3 × 10^4^ cells, and the macrophage samples were collected at 1 × 10^4^ cells for analysis.

### 2.12. Western Blot

The spleen cells and THP-1-derived M1 macrophages were lysed with the RIPA lysis buffer, and protein concentrations were measured using a BCA protein assay kit according to the manufacturer’s instructions. Proteins were separated by electrophoresis on 12.5% polyacrylamide gels and transferred to polyvinylidene fluoride (PVDF) membranes. The membranes were blocked with a rapid blocking buffer, followed by incubation with primary antibodies at 4 °C for 12 h and secondary antibodies at 37 °C for 1 h. The primary antibodies included anti-LC3 (1:2000), anti-P62 (1:1000), anti-Parkin (1:1000), anti-Pink1 (1:1000), anti-GAPDH (1:50,000), and anti-β-actin (1:4000). The secondary antibodies included horseradish peroxidase (HRP)-conjugated goat anti-rabbit IgG (H + L) (ZB-2301, ZSGB-BIO) and HRP-conjugated goat anti-mouse IgG (H + L) (ZB-2305, ZSGB-BIO). The PVDF membranes were imaged using a Tanon 5200 Multi imaging system (Tanon Science & Technology Co., Ltd., Shanghai, China). Band intensity was analyzed using the ImageJ software (2.1.0/1.53c).

### 2.13. ELISA

In this study, enzyme-linked immunosorbent assay (ELISA) kits for human IL-1β, IL-10, TGF-β, and TNF-α were used. Supernatants from the M1 macrophages treated with PBS, NaF, and NaF + CQ were collected and analyzed according to the instructions of the respective ELISA kits. The concentrations of IL-1β, IL-10, TGF-β, and TNF-α were calculated using standard curves, with all the (R^2^) values exceeding 0.99.

### 2.14. Mitochondrial Membrane Potential (MMP) Detection

MMP was evaluated using the JC-1 mitochondrial membrane potential assay kit according to the manufacturer’s instructions. Detection was performed using a confocal microscope (LSM 800, Zeiss, Germany) and a flow cytometer (BD Melody, BD Biosciences, San Jose, CA, USA). Changes in mitochondrial membrane potential were determined by measuring the fluorescence intensity of the JC-1 aggregates and monomers.

### 2.15. Statistical Analysis

Statistical analysis and graphing were performed using the SPSS 26.0 software and GraphPad Prism 10.1.1 (GraphPad Software, LLC. San Diego, CA, USA). For normally distributed continuous data, comparisons between two groups were performed using a t-test, and comparisons among multiple groups were performed using one-way analysis of variance (ANOVA), with data presented as mean ± standard deviation (SD). For non-normally distributed data, the Mann–Whitney U test was used, and data are presented as median (P25, P75). Categorical variables were compared using the chi-square test, and count data are presented as proportions. Spearman’s rank correlation coefficient was used to analyze correlations for non-normally distributed data. Statistical significance was set at * *p* < 0.05, ** *p* < 0.01, *** *p* < 0.001, and **** *p* < 0.0001. All the hypothesis tests were two-tailed, with *p* < 0.05 considered statistically significant.

## 3. Results

### 3.1. Population Epidemiological Survey

In this study, the fluoride concentrations in drinking water from Wujian Village, Hongqing Village, Wujing Village, Hongguang Village, and Pingfang Village in Zhaodong City, Heilongjiang Province, were measured at 0.570 mg/L, 0.764 mg/L, 1.187 mg/L, 1.342 mg/L, and 2.027 mg/L, respectively. As shown in Table 1, a total of 249 participants (101 males and 148 females) were included, with an average age of 58.14 ± 10.76 years, a median urinary fluoride concentration of 1.19 mg/L, and an average BMI of 24.23 ± 3.35 kg/m^2^. There were no statistically significant differences in urinary fluoride concentrations across various demographic characteristics and lifestyle factors (education level, household income, smoking status, drinking habits, or annual frequency of colds) (*p* > 0.05). Based on the tertiles of urinary fluoride concentrations (P33.3 and P66.7), the participants were divided into three groups: urinary fluoride ≤ 0.897 mg/L, urinary fluoride 0.897–1.432 mg/L, and urinary fluoride > 1.432 mg/L. The geometric mean urinary fluoride concentrations for these groups were 0.62 mg/L, 1.15 mg/L, and 1.97 mg/L, respectively. As shown in Table 2, there were no significant differences in age, gender, education level, household income, smoking, or drinking distribution among the different urinary fluoride groups (*p* > 0.05). However, there was a statistically significant difference in BMI among the groups (*p* < 0.05), with the urinary fluoride > 1.432 mg/L group having a significantly lower BMI compared to the urinary fluoride 0.897–1.432 mg/L group. Chi-square test analysis revealed no significant statistical association between urinary fluoride levels and the annual frequency of colds (*p* > 0.10). However, a trend test indicated a significant linear trend between urinary fluoride levels and the frequency of colds (*p* < 0.05). As urinary fluoride concentrations increased, the proportion of individuals experiencing more than four colds per year also showed an upward trend, suggesting a potential link between fluoride exposure and immune status.

### 3.2. Effects of Fluoride on Monocyte Subsets in Human Blood

**Figure 1:**
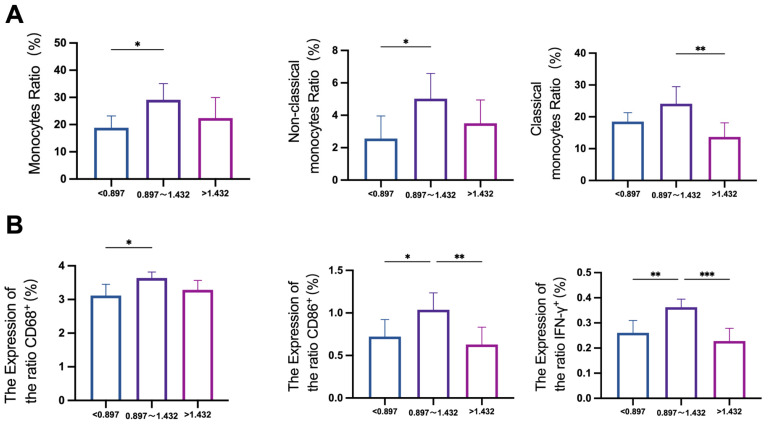
Effects of fluoride exposure on monocytes and related markers in blood. (**A**) Mass cytometry was used to detect the changes in monocytes and non-classical monocytes in the blood of different urine fluoride groups. (**B**) The changes in macrophage marker CD68, M1 macrophage marker CD86, and inflammatory cytokine IFN-γ in the blood of different urine fluoride groups were detected by mass spectrometry flow cytometry. * *p* < 0.05, ** *p* < 0.01, and *** *p* < 0.001.

Monocytes are a highly heterogeneous population of cells in the blood that play a critical role in innate immune responses to inflammation and are involved in antigen presentation during adaptive immunity [45]. In this study, CD68 was used as a macrophage marker, cluster of differentiation 86 (CD86) as an M1 macrophage marker, and cluster of differentiation 206 (CD206) as an M2 macrophage marker. As shown in Figure 1A, compared to the control group, the proportion of monocytes(CD11b^+^CD14^+^) in peripheral blood significantly increased in the urinary fluoride 0.897–1.432 mg/L group. Specifically, the proportion of non-classical monocytes (CD14^lo^CD16^+^) showed a significant increase (*p* < 0.05), while the proportion of classical monocytes(CD14^+^CD16^-^) exhibited an upward trend in the urinary fluoride 0.897–1.432 mg/L group but a downward trend in the urinary fluoride > 1.432 mg/L group. As shown in Figure 1B, the expression of the macrophage marker CD68 and the M1 macrophage marker CD86 in peripheral blood increased in the urinary fluoride 0.897–1.432 mg/L group (*p* < 0.05) but decreased in the urinary fluoride > 1.432 mg/L group. The pro-inflammatory cytokine IFN-γ showed elevated expression in peripheral blood cells of the urinary fluoride 0.897–1.432 mg/L group (*p* < 0.01) but decreased expression in the urinary fluoride > 1.432 mg/L group. The population study results indicate that the levels of monocytes and pro-inflammatory factors in peripheral blood were significantly elevated in the urinary fluoride 0.897–1.432 mg/L group, suggesting that inflammatory responses may be associated with fluoride exposure. In the urinary fluoride 0.897–1.432 mg/L group, an inflammatory response may occur, while a compensatory response appears to take place in the urinary fluoride > 1.432 mg/L group.

### 3.3. Verification of the Rat Fluorosis Model

**Figure 2:**
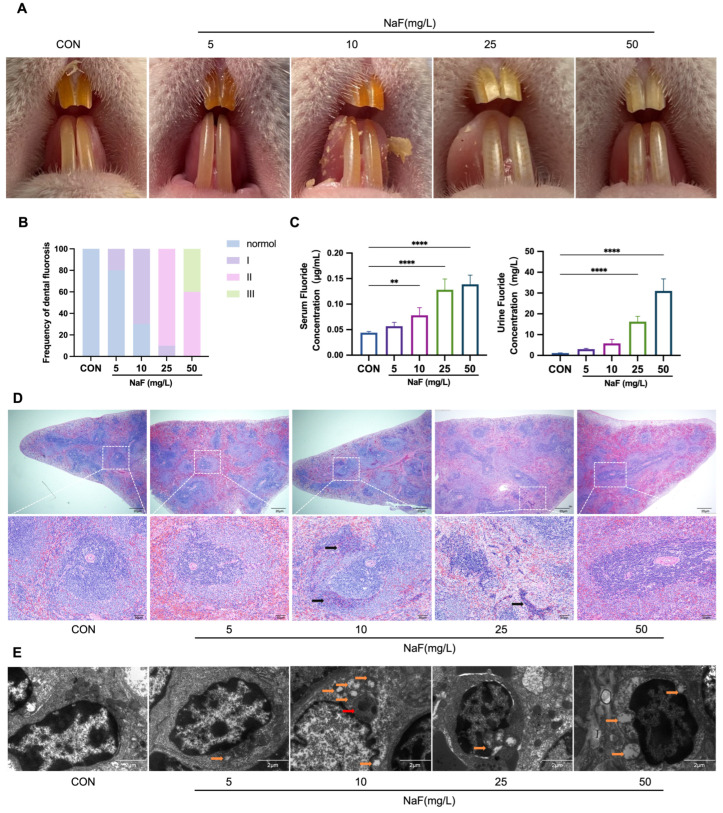
Fluorosis and the effects of fluoride on the spleen in the rats: (**A**) Representative images of dental fluorosis in the rats from the different fluoride treatment groups. (**B**) Frequency of varying degrees of dental fluorosis in the rats from each fluoride treatment group. (**C**) Changes in fluoride concentrations in the blood and urine of the rats from the different fluoride treatment groups. (**D**) Representative H&E-stained microscopic images of the spleen tissue from the rats in the different fluoride treatment groups.Black arrows indicate inflammatory cell infiltration. Scale bar: 20 µm (above) and 50 µm (below). (**E**) Ultrastructural changes in the spleen tissue from the rats in the different fluoride treatment groups (orange arrows indicate damaged mitochondria; red arrows indicate autophagosomes). ** *p* < 0.01 and **** *p* < 0.0001.

Based on findings from the population study, an animal model was developed to further investigate the effects of fluoride on macrophages. Dental fluorosis is one of the earliest clinical manifestations of excessive fluoride exposure [46]. At the end of the experiment, the teeth of the control group rats exhibited normal development, with a glossy surface and good translucency. In the low-fluoride-treated group, the teeth showed almost no change in glossiness but displayed small white streaks. In contrast, the teeth of the rats in the moderate- and high-fluoride-treated groups were unevenly pigmented and appeared chalky white (Figure 2A). Exposure to varying fluoride concentrations results in different degrees of dental fluorosis in rats, with symptoms becoming more severe and the incidence of dental fluorosis increasing as the fluoride concentration in drinking water rises. All the rats in the moderate- and high-fluoride-treated groups exhibited varying degrees of dental fluorosis symptoms (Figure 2B). Additionally, fluoride ion concentrations in the rat blood and urine were positively correlated with fluoride levels in drinking water (Figure 2C). These results confirm the successful establishment of a rat fluorosis model.

### 3.4. Fluoride Causes Spleen Damage and Induces Inflammatory Responses

To investigate whether fluoride affects immune organs, hematoxylin and eosin (H & E) staining and transmission electron microscopy (TEM) were performed on the spleen. As shown in Figure 2D, the spleens of the control group displayed normal structural features, including lymphatic nodules and periarterial lymphatic sheaths within the white pulp. Lymphocytes were densely packed and deeply stained around the central artery, with a clearly defined marginal zone. With increasing fluoride treatment, the marginal zone of the rats’ spleen gradually expanded, and its boundaries became indistinct. Inflammatory cell infiltration was observed in the spleens of the rats in the 10 mg/L and 25 mg/L fluoride-treated groups. The TEM analysis revealed that in the control group, the cytoplasm and nucleus were closely apposed, and mitochondria appeared elongated or kidney-shaped with well-defined cristae. In all the rats of the NaF-treated groups, varying degrees of mitochondrial swelling and the disappearance of mitochondrial cristae were observed. Autophagosomes were predominantly detected in the 10 mg/L fluoride-treated group, which may indicate that at lower fluoride concentrations, cells initiate autophagy as an adaptive response to fluoride-induced mitochondrial stress and damage. Nuclear pyknosis was evident in the 25 mg/L fluoride-treated group (Figure 2E). The results indicate that low fluoride leads to cellular damage in the rat spleen, with particularly severe mitochondrial damage, and alters the morphological structure of the spleen. Inflammatory responses were observed in both the low-fluoride and moderate-fluoride-treated groups.

### 3.5. Low Fluoride Increases the M2/M1 Ratio in the Spleen

**Figure 3:**
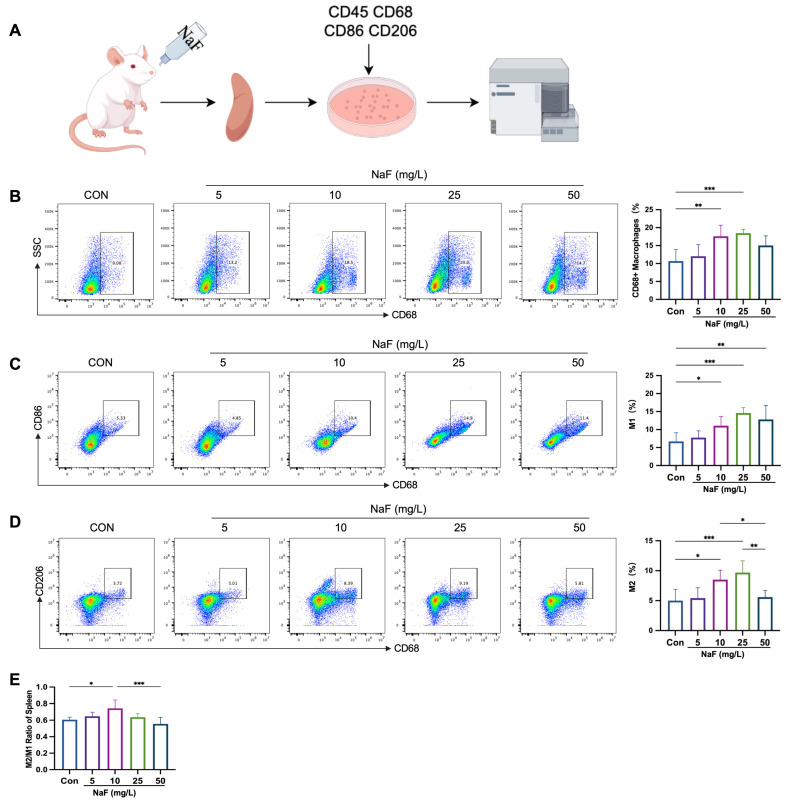
Low fluoride increases the M2/M1 ratio in the spleen: (**A**) Flow cytometry processing workflow for the splenic cells from the rats. (**B**) The proportion of CD68⁺ macrophages (M0) in the spleens of the rats exposed to different fluoride doses, detected by flow cytometry. (**C**) The proportion of CD68⁺CD86⁺ macrophages (M1) in the spleens of the rats exposed to different fluoride doses, detected by flow cytometry. (**D**) The proportion of CD68⁺CD206⁺ macrophages (M2) in the spleens of the rats exposed to different fluoride doses, detected by flow cytometry. (**E**) M2/M1 ratio in the spleens of the rats exposed to different fluoride doses, detected by flow cytometry. * *p* < 0.05, ** *p* < 0.01 and *** *p* < 0.001.

A population study revealed an increase in monocytes and macrophage-related markers in individuals with low fluoride treatment. To investigate whether NaF affects the balance of macrophage subtypes, we performed flow cytometry on the splenic cells from the rats (the processing flow is shown in Figure 3A). The results showed that the proportion of M0 macrophages (CD68⁺) increased in all the fluoride treatment groups compared to the control group, with significant increases observed in the 10 mg/L and 25 mg/L NaF-treated groups (*p* < 0.05), while a decreasing trend was noted in the high fluoride-treated group (Figure 3B). The proportion of M1 macrophages (CD68⁺CD86⁺) significantly increased in the 10 mg/L, 25 mg/L, and 50 mg/L NaF-treated groups (*p* < 0.05) (Figure 3C). Similarly, the proportion of M2 macrophages (CD68⁺CD206⁺) increased in the 10 mg/L and 25 mg/L NaF-treated groups (*p* < 0.05), but showed a decreasing trend in the 50 mg/L NaF-treated group (Figure 3D). Notably, low-dose NaF elevated the M2/M1 ratio in the spleen, with the most significant changes observed in the 10 mg/L NaF treatment group (Figure 3E). These results suggest that low fluoride exposure can trigger a pro-inflammatory response in the body, which is accompanied by the activation of anti-inflammatory pathways under long-term NaF treatment. Although the proportion of both M1 and M2 macrophages exhibited increasing trends, the significantly elevated M2/M1 ratio in the 10 mg/L NaF group may indicate the onset of an inflammation resolution phase. Whether the increase in M2 macrophages is due to the phenotypic transition from pro-inflammatory M1 macrophages to anti-inflammatory M2 macrophages requires further experimental verification.

### 3.6. Fluoride Activates the Mitochondrial Autophagy Pathway in the Spleen

**Figure 4:**
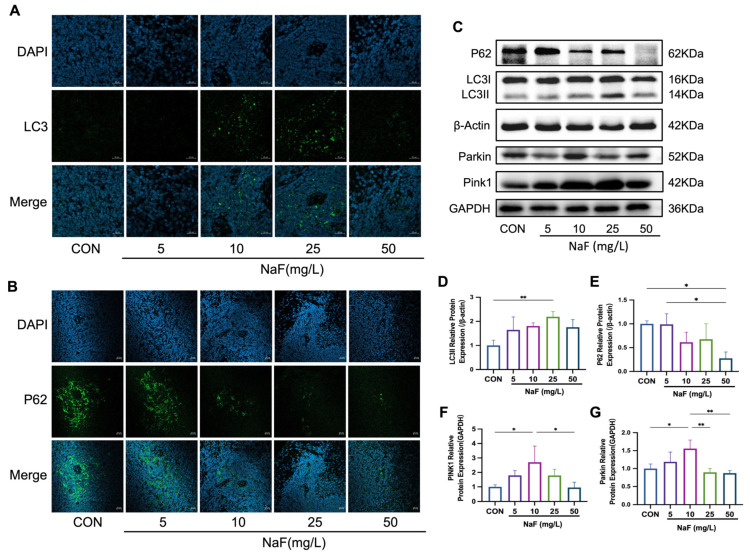
Fluoride activates the mitochondrial autophagy pathway in the spleen: (**A**,**B**) Expression of autophagy markers LC3 and P62 in the five treatment groups observed using confocal laser scanning microscopy. Scale bar: 20 µm. (**C**) Western blot analysis. (**D**–**G**) Relative expression levels of mitochondrial autophagy-related proteins P62, Parkin, PINK1, and LC3. * *p* < 0.05 and ** *p* < 0.01. Original images can be found in Figure S1.

In the spleen ultrastructural sections, autophagosomes were observed in the low-fluoride treatment group, along with varying degrees of mitochondrial damage in all the fluoride treatment groups. Based on these findings, we hypothesized that fluoride induces autophagy, potentially involving mitophagy. To validate this, we conducted immunofluorescence and Western blot (WB) experiments on the rat spleen tissues. Immunofluorescence analysis revealed increased LC3 expression in the 10 mg/L and 25 mg/L NaF-treated groups, while P62 expression decreased with higher fluoride concentrations (Figure 4A,B). The WB results showed a decreasing trend in the relative expression of P62 with increasing fluoride treatment, with a significant reduction in the 50 mg/L NaF-treated group (*p* < 0.05). Conversely, LC3II expression exhibited an increasing trend in the fluoride treatment groups, peaking significantly in the 25 mg/L NaF-treated group (*p* < 0.05) (Figure 4D,E). These changes in the expression of autophagy markers indicate that fluoride activates the autophagic pathway in the spleen. PINK1, a sensor of mitochondrial dysfunction, recruits Parkin to facilitate the degradation of damaged mitochondria via mitophagy [47]. Our findings showed that PINK1 and Parkin expression increased significantly in the low-fluoride treatment group (*p* < 0.05) but exhibited a decreasing trend in the medium and high-fluoride groups (Figure 4F,G). In summary, these results suggest that NaF activates the mitochondrial autophagy signaling pathway in the spleen.

### 3.7. Mitochondrial Autophagy Mediates Fluoride-Induced Alterations in Macrophage M2/M1 Balance

**Figure 5:**
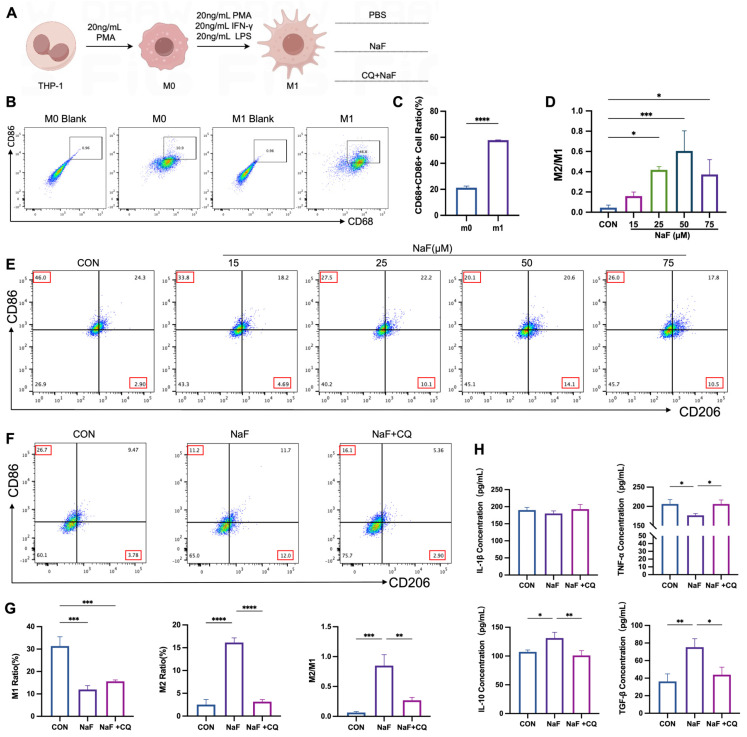
NaF induces an M2/M1 ratio shift through the activation of the mitochondrial autophagy pathway: (**A**) Induction of M1 macrophages derived from THP-1 cells and subsequent intervention procedures. (**B**,**C**) Validation of the induction of M1 macrophages using flow cytometry. (**D**,**E**) Effects of different fluoride concentrations on the M2/M1 ratio. (**F**,**G**) Flow cytometry analysis of the proportions of M1 and M2 macrophages, as well as the changes in the M2/M1 ratio, in the control group, NaF-treated group, and CQ + NaF-treated group. (**H**) ELISA analysis of M1-associated cytokines (IL-1β and TNF-α) and M2-associated cytokines (IL-10 and TGF-β) in the control, NaF, and CQ + NaF treatment groups. * *p* < 0.05, ** *p* < 0.01, *** *p* < 0.001, and **** *p* < 0.0001.

In the animal experiments, fluoride induced an inflammatory response in the spleen, accompanied by an increase in macrophage numbers, particularly M2 macrophages, resulting in an elevated M2/M1 ratio. Moreover, the mitochondrial autophagy pathway was activated in the spleen tissues. Based on these findings, we hypothesized that fluoride might regulate the M2/M1 macrophage balance through mitochondrial autophagy. To test this hypothesis, we constructed an in vitro inflammatory model of M1 macrophages and treated them with NaF (the processing flow is shown in Figure 5A). The induction efficiency of the M1 macrophages derived from THP-1 cells was validated by flow cytometry, and macrophage polarization was assessed. The results showed a significant increase in M1 macrophages compared to M0 macrophages after induction (Figure 5B,C). To determine the optimal concentration of NaF, the M1 macrophages were treated with various doses of NaF (0, 15, 25, 50, and 75 µM). As shown in Figure 5D,E, the 50 µM NaF-treated group exhibited the most significant increase in the M2/M1 ratio, and this concentration was selected for subsequent experiments. CQ is a weakly basic compound that can penetrate cell membranes and increase lysosomal pH, thereby inhibiting the fusion of autophagosomes with lysosomes or reducing lysosomal activity, which prevents the degradation of autophagosomal contents and blocks autophagic flux. Animal experiments have suggested that NaF activates the autophagy pathway. To investigate whether NaF-induced polarization of M1 macrophages to M2 macrophages is mediated by mitophagy, the M1 macrophages were pretreated with the autophagy inhibitor chloroquine (CQ) followed by the NaF treatment, and flow cytometry was performed for analysis. The results showed that NaF significantly reduced the proportion of M1 macrophages (*p* < 0.001) while increasing the proportion of M2 macrophages (*p* < 0.0001), leading to a significant elevation in the M2/M1 ratio (*p* < 0.001). These effects were reversed upon the CQ treatment (Figure 5F,G). The ELISA results further confirmed that NaF t reduced the secretion of the M1-associated cytokine TNF-α (*p* < 0.05), which was restored by CQ intervention. Conversely, the expression of the M2-associated cytokines IL-10 and TGF-β increased following the NaF treatment but was reversed by the CQ treatment (Figure 5H). These results indicate that mitochondrial autophagy is involved in the process by which NaF influences the M2/M1 macrophage balance.

### 3.8. Fluoride Causes Mitochondrial Damage in Macrophages

**Figure 6:**
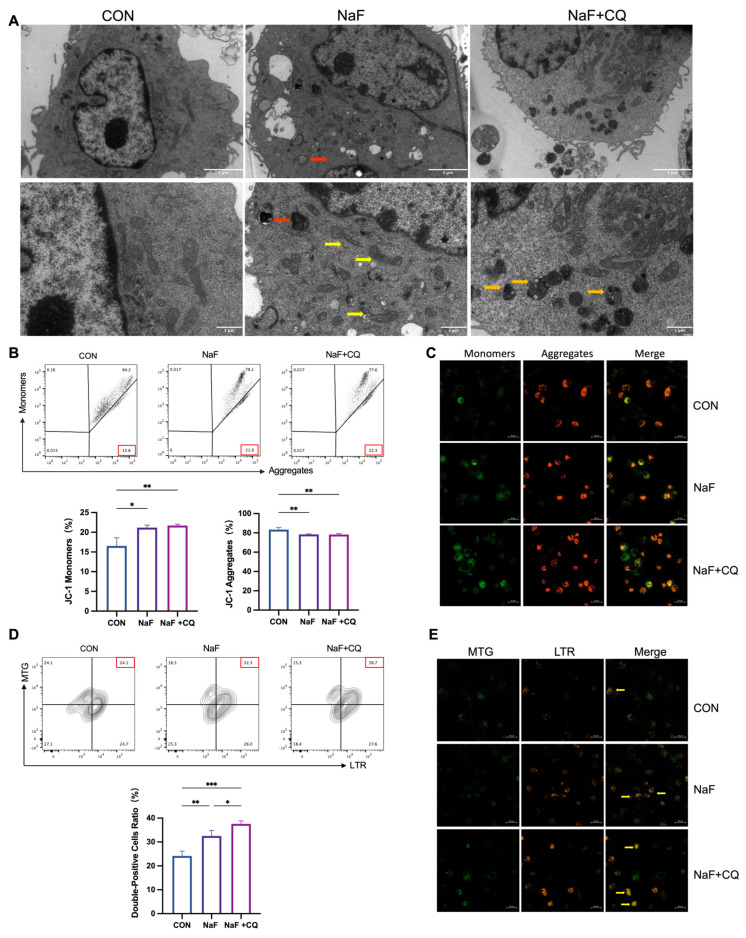
Fluoride induces mitochondrial damage and mitochondrial autophagy in macrophages. (**A**) Representative transmission electron microscopy images of the THP-1-derived M1 macrophages treated with PBS, 50 µM NaF, and 20 µM CQ + 50 µM NaF for 24 h (red arrows indicate mitophagosomes; yellow arrows indicate damaged mitochondria; orange arrows indicate secondary lysosomes. Scale bar: 3 µm). (**B**) MMP levels in the THP-1-derived M1 macrophages were measured using flow cytometry. (**C**) Changes in mitochondrial membrane potential were observed after JC-1 staining using confocal laser scanning microscopy. JC-1 monomers (green fluorescence) indicate damaged mitochondria, while JC-1 aggregates (red fluorescence) represent normal mitochondria. Scale bar: 20 µm (**D**) Mitochondrial-lysosome colocalization in the THP-1-derived macrophages was detected using flow cytometry. (**E**) Mitochondria–lysosome colocalization was observed by confocal laser scanning microscopy (yellow arrows indicate colocalized mitochondria and lysosomes. Scale bar: 20 µm). * *p* < 0.05, ** *p* < 0.01, and *** *p* < 0.001.

To further investigate the mechanism of fluoride-induced mitochondrial autophagy, the M1 macrophages were treated with NaF, and several evaluations were performed: MMP was assessed to evaluate mitochondrial status; TEM was used to observe the mitochondrial structure and the formation of mitophagosomes; and colocalization of mitochondria and lysosomes was analyzed using specific probes to examine their interaction. As shown in Figure 6A, in the control group, the mitochondria displayed intact structures, with an oval or elongated shape, smooth and continuous outer membranes, and clearly visible cristae. Only a small number of primary lysosomes were scattered in the cytoplasm. In the NaF-treated group, cytoplasmic vacuoles appeared, mitochondria shrank and became oblate, outer membranes were blurred, and membrane-enclosed mitochondrial structures as well as autophagolysosomes were observed. In the NaF + CQ-treated group, secondary lysosomes significantly increased, and mitochondria showed pronounced shrinkage. These observations suggest that NaF induces mitochondrial autophagy to eliminate damaged mitochondria. In the presence of CQ, the accumulation of secondary lysosomes and damaged mitochondria indicates that autophagic flux was inhibited. A stable MMP is essential for oxidative phosphorylation and ATP production, which are prerequisites for maintaining normal cellular functions. When MMP is high, JC-1 aggregates in the mitochondrial matrix to form J-aggregates, emitting red fluorescence. When MMP is low, JC-1 remains as monomers, emitting green fluorescence. Using JC-1 probes, MMP in M1 macrophages was analyzed. Flow cytometry results showed a significant decrease in MMP in NaF-treated and CQ + NaF-treated M1 macrophages (*p* < 0.05) (Figure 6B). Confocal microscopy revealed a marked increase in green fluorescence, indicating reduced MMP, consistent with the flow cytometry findings (Figure 6C). Dual staining with MitoTracker Green (MTG) and LysoTracker Red (LTR) was used for fluorescence localization of mitochondrial autophagy. The flow cytometry results indicated an increased proportion of cells with the colocalization of mitochondria and lysosomes in the NaF-treated group (*p* < 0.01), which was further amplified in the NaF + CQ group (Figure 6D). Additionally, immunofluorescence staining showed enhanced colocalization signals and increased fluorescence intensity of mitochondria and lysosomes in both the NaF and NaF + CQ-treated groups (Figure 6E). These results indicate the occurrence of mitochondrial autophagy, and the further increase in colocalization signals in the presence of CQ suggests impaired mitochondrial degradation due to autophagic flux blockage.

### 3.9. Fluoride Activates the PINK1/Parkin-Mediated Mitophagy Pathway

**Figure 7:**
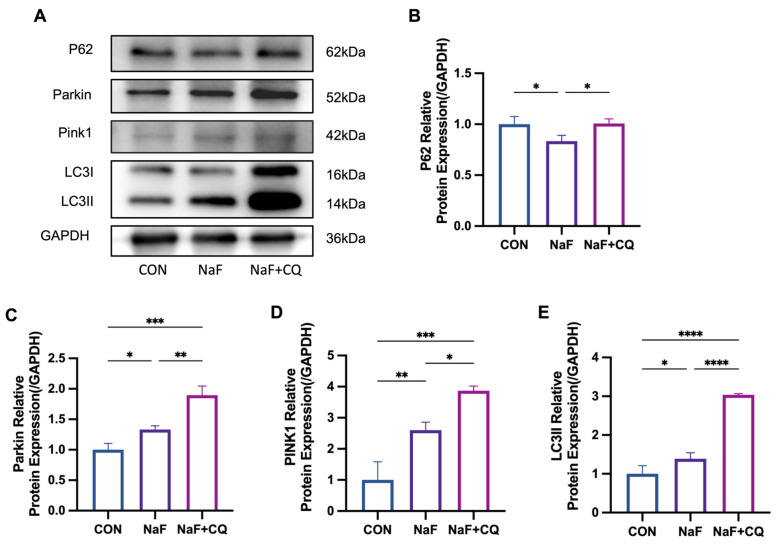
Changes in the relative expression of PINK1/Parkin-mediated mitophagy signaling pathway proteins in the spleen detected by Western blotting: (**A**) Western blotting. (**B**–**E**) Relative expression of P62, Parkin, PINK1, and LC3 proteins. * *p* < 0.05, ** *p* < 0.01, *** *p* < 0.001, and **** *p* < 0.0001. Original images can be found in Figure S2.

To investigate the role of mitophagy in fluoride-induced macrophage polarization, autophagy inhibitors (CQ) were added before fluoride intervention, and mitochondrial autophagy-related proteins were detected in all the groups. The Western blotting results showed that in the M1 macrophages, the expression of P62 was significantly reduced in the NaF-treated group compared to the control group (*p* < 0.05), whereas its expression increased following the CQ treatment. (Figure 7B). The expression of LC3II was elevated after the NaF treatment (*p* < 0.05), and its expression was further significantly increased after the CQ treatment (*p* < 0.0001) (Figure 7E). Additionally, the expression of PINK1 and Parkin were significantly elevated in the NaF-treated group compared to the control group (*p* < 0.05). Moreover, the NaF + CQ-treated group exhibited a more pronounced increase in their expression levels compared to the control group. (*p* < 0.001) (Figure 7C,D). These results indicate that mitophagy was activated after the NaF treatment, and after the CQ intervention, damaged mitochondria or other organelles could not be properly degraded, blocking autophagic flux, and leading to a further increase in the expression of these proteins.

## 4. Discussion

The effectiveness of fluoride removal from drinking water has been significant in various countries [39,40,41], with the number of areas with high fluoride exposure gradually decreasing. However, areas with low to moderate fluoride exposure have notably increased, which has raised concerns among researchers regarding the potential health impacts of low-level fluoride exposure. Previous population studies have indicated that low fluoride exposure can lead to changes in immune cell subsets, thereby impairing immune function [48]. However, the impact of low fluoride exposure on macrophage polarization remains to be explored. According to the World Health Organization (WHO) recommended standards, the maximum allowable fluoride concentration in drinking water is 1.5 mg/L [49]. This study selected five villages in Zhaodong City, Heilongjiang Province, China, with an average fluoride concentration range of 0.570–2.027 mg/L in drinking water, covering both low and high fluoride exposure levels. Therefore, this population can be used to investigate the potential relationship between long-term low fluoride exposure and the immune system. This study found that individuals experiencing more than four colds per year had higher urinary fluoride levels, although the difference was not statistically significant. Further analysis after grouping by urinary fluoride concentration revealed a weak positive correlation between urinary fluoride levels and the frequency of colds. Specifically, as urinary fluoride concentrations increased, the proportion of individuals experiencing more than four colds per year showed an upward trend. Compared to the group with urinary fluoride concentrations < 0.897 mg/L, the group with urinary fluoride concentrations of 0.897–1.432 mg/L exhibited a significant increase in the proportion of monocytes in peripheral blood, along with the elevated expression of pro-inflammatory markers (CD68, CD86, and IFN-γ). This finding suggests that even low levels of fluoride exposure may trigger inflammatory responses in the body. Notably, the geometric mean urinary fluoride concentration in the 0.897–1.432 mg/L group was 1.15 mg/L, which is below the recommended urinary fluoride safety threshold of ≤1.6 mg/L for adults aged 18 and above, as specified in the WS/T 10023-2024: Safety Guideline for Urinary Fluoride in Populations issued by the National Health Commission of China [50]. This guideline establishes reference values for urinary fluoride concentrations across different age groups based on health risk assessments, aiming to prevent chronic fluoride toxicity. However, although the urinary fluoride levels in this group remain within the so-called “safe” range, individuals already exhibited signs of monocyte-driven inflammatory activation. This indicates that the current safety threshold may not fully capture the potential immunotoxic risks associated with chronic low-level fluoride exposure.

Based on findings from population studies, we established an animal model and adjusted the dose differences between humans and rodents using the body surface area dose conversion formula [51]. The selected fluoride concentrations (5, 10, 25, and 50 mg/L NaF) corresponded to human equivalent doses of approximately 0.81, 1.61, 4.03, 8.06, and 16.12 mg/L, respectively. Additionally, Zhou et al. [7] defined low fluoride exposure in rats as fluoride ion concentrations ≤ 20.9 mg/L in drinking water. Our experimental design covers both low and high fluoride exposure ranges, thereby laying the groundwork for further mechanistic investigations.

The spleen is the largest peripheral immune organ in the body and plays a crucial role in immune regulation [52]. Assessing its pathological changes is key to understanding immune function. In this study, morphological changes such as inflammatory cell infiltration, nuclear condensation, mitochondrial swelling, and the disappearance of mitochondrial cristae were observed in spleen sections stained with H&E from the low-to-moderate fluoride-treated group. This finding is consistent with other studies. For example, Qiao et al. found that high fluoride (100 mg/L) led to inflammatory cell infiltration in the rat spleens [14]. Kuang et al. reported that low fluoride (12 mg/L and 24 mg/L) caused a reduction in spleen volume, induced spleen cell apoptosis, and resulted in mitochondrial dysfunction [53]. Additionally, Zhao [34] and Liang [32] found that high fluoride caused mitochondrial damage and autophagy in hepatocytes and stromal cells of the testis in mice. In the present study, autophagosomes were observed in the spleens of the rats treated with 10 mg/L fluoride. These findings suggest that the morphological structure of the spleen begins to exhibit damage under low fluoride conditions.

Numerous studies have demonstrated that fluoride exhibits a concentration-dependent, bidirectional regulatory effect on immune and inflammatory responses. At low concentrations (1–10 µM), fluoride can modulate the expression and activity of enzymes such as COX-1 and COX-2, which are involved in initiating and propagating inflammation [29]. High concentrations of fluoride (>50 mg/L) have pronounced immunotoxic effects, inducing lymphocyte apoptosis and causing atrophy of immune organs like the thymus and spleen [54,55].

Previous research has indicated that high fluoride levels promote the polarization of macrophages toward the pro-inflammatory M1 phenotype [26,27]. In our current study, we observed that low fluoride concentrations increased the numbers of both M1 and M2 macrophages; however, the M1/M2 ratio suggested a shift toward the anti-inflammatory M2 phenotype. The rise in M1 macrophages indicates the presence of inflammatory damage, while the increase in M2 macrophages may reflect a compensatory anti-inflammatory response by the body. This suggests that under low fluoride exposure, the immune system attempts to establish a dynamic balance between inflammation and repair, showcasing its adaptive regulatory capacity. However, this compensatory mechanism might also predispose tissues to chronic pathological changes, such as fibrosis. The elevated M2 macrophage proportion could result from the phenotypic switching of M1 macrophages at sites of injury [56,57]. In the group exposed to high fluoride concentrations (50 mg/L), we noted a decreasing trend in M2 macrophage proportions, aligning with findings by Du et al. [27], who reported that high fluoride intake impairs fracture healing by inhibiting M2 macrophage polarization. The underlying mechanisms of this phenomenon remain unclear and warrant further investigation.

The regulation of macrophages is closely associated with mitochondrial autophagy. Previous studies have demonstrated that IL-25 and arsenic can activate oxidative stress, damage mitochondria, and trigger mitochondrial autophagy, thereby inducing the polarization of macrophages from M1 to M2 [58,59]. Similarly to arsenic, NaF has been shown to increase the production of ROS, induce oxidative stress, reduce mitochondrial membrane potential, and cause mitochondrial damage [32,33,60,61]. Our study revealed that low fluoride caused mitochondrial damage in the spleens of the rats and induced mitochondrial autophagy in splenic cells through the PINK1/Parkin signaling pathway. This finding is consistent with the study by Liang et al. [32], who reported that fluoride induces PINK1/Parkin-mediated mitochondrial autophagy in stromal cells of the testis. Using an in vitro inflammatory model of M1 macrophages, we found that NaF led to mitochondrial damage and mitochondrial autophagy, promoting the polarization of M1 macrophages to M2 macrophages and increasing the M2/M1 ratio. Pretreatment with CQ inhibited NaF-induced mitochondrial autophagy, and this inhibition reversed the NaF-induced polarization of M1 macrophages to M2 macrophages. These results are consistent with those of Ge [62] and Wang [63], who also found that autophagy inhibited M1 polarization and promoted M2 polarization in hepatic macrophages. Therefore, low fluoride may promote the polarization of splenic macrophages from M1 to M2 through mitochondrial autophagy, which could explain the observed increase in M2 macrophages in the spleens of animals in this study.

Macrophage polarization plays a pivotal role in regulating the progression and resolution of inflammation [26,64]. Recent advancements in ribozyme-based technologies offer novel avenues for the post-transcriptional modulation of macrophage polarization, enabling precise adjustments to immune responses. Ribozymes are catalytic RNA molecules capable of specifically cleaving target mRNAs, thereby downregulating the proteins involved in immune activation or suppression pathways [65]. For instance, targeting the key regulators associated with M1 polarization, such as HIF-1α [66], or those linked to M2 polarization, like PPARγ [67], can effectively influence macrophage polarization states.

Integrating ribozyme-based strategies with the dynamics of macrophage polarization presents a promising therapeutic approach to mitigate immunotoxic effects induced by environmental exposures, such as fluoride. However, the feasibility, specificity, and safety of ribozyme applications in vivo require further investigation, particularly in the context of chronic low-dose toxicant exposure. Future research should focus on optimizing ribozyme delivery systems to enhance their stability and efficiency within target cells. Achieving precise control over macrophage polarization through ribozyme technology could provide effective interventions for inflammation-related diseases.

This study investigated the role of mitochondrial autophagy in NaF-induced macrophage polarization through animal experiments and in vitro cellular models. However, this study still has several limitations. First, while the animal model simulated long-term low fluoride by providing water containing low fluoride concentrations over 12 weeks, the absence of natural infections and environmental immune stimulation under sterile conditions may not accurately reflect the effects of low-dose chronic exposure or the complex microenvironment on immune responses. Second, macrophage polarization in the in vitro studies was conducted under highly controlled conditions, lacking the intricate regulation of immune cells, cytokines, metabolites, and signaling pathways present in the in vivo microenvironment. Consequently, these models may not fully replicate the dynamic polarization process observed in vivo. Additionally, in vitro studies often rely on single stimulation factors (e.g., LPS, PMA, or IFN-γ) and short time windows, which cannot simulate the dynamic changes driven by multiple signals in vivo. Therefore, while these findings offer valuable insights, further population-based studies are required to validate the extrapolation of these experimental results to human populations.

## 5. Conclusions

This study demonstrates that low-level fluoride has significant impacts on the immune system. Specifically, low fluoride markedly increased the number of monocytes in the blood and significantly promoted their pro-inflammatory polarization, potentially contributing to inflammatory responses. Additionally, low fluoride led to morphological and structural changes in the spleen, including inflammatory cell infiltration and mitochondrial swelling. Mechanistic investigations further revealed that low fluoride can induce mitophagy by activating the PINK1/Parkin signaling pathway and promoting the polarization of M1 macrophages to M2 macrophages. These findings not only elucidate the potential mechanisms underlying the detrimental effects of low fluoride (<1.5 mg/L) on macrophages and the immune system but also provide scientific evidence for establishing more precise standards for fluoride exposure in drinking water.

## Figures and Tables

**Table 1:** General information of the participants.

Variable	Sample	Urinary Fluoride Concentration (mg/L) ^c^	t/F	*p*
Total	249	1.19 (0.79, 1.58)		
Age(years) ^b^	58.14 ± 10.76			
Sex ^a^			−0.55	0.43
Male	101 (40.6%)	1.08 (0.82, 1.58)		
Female	148 (59.4%)	1.23 (0.78, 1.58)		
BMI(kg/m^2^) ^b^	24.23 ± 3.35			
Educational level ^a^			1.36	0.26
Primary school	171 (69%)	1.26 (0.82, 1.62)		
Junior high school	64 (26%)	1.00 (0.74, 1.42)		
Senior high school	13 (5%)	1.10 (0.72, 1.57)		
Annual household income ^a^			0.57	0.57
<1.2 w	90 (36%)	1.15 (0.81, 1.53)		
1.2–2 w	59 (24%)	1.23 (0.82, 1.58)		
>2 w	100 (40%)	1.19 (0.79, 1.61)		
Smoke ^a^			0.61	0.43
YES	71 (29%)	1.18 (0.82, 1.56)		
NO	178 (71%)	1.20 (0.78, 1.58)		
Drink ^a^			3.25	0.07
YES	74 (30%)	1.03 (0.82, 1.50)		
NO	175 (70%)	1.24 (0.77, 1.59)		
Colds(every year) ^a^			−1.97	0.07
0–4 times	236 (95%)	1.18 (0.77, 1.58)		
More than 4 times	13 (5%)	1.47 (0.96, 1.72)		

^a^ categorical variables are expressed as numbers (percentages). ^b^ mean ± standard deviation for continuous variables. ^c^ continuous variables are represented by the median (P25,P75).

**Table 2:** Basic information of different urinary fluoride groups.

Variable	Urinary Fluoride Concentration (mg/L)			F/χ^2^	*p*
	<0.897	>0.897–≤1.432	>1.432		
Urinary fluoride level ^a^	0.65 (0.50, 0.79)	1.19 (0.99, 1.33)	1.77 (1.58, 2.15)	378.93	0.00
Age(years) ^a^	56.89 ± 10.49	57.98 ± 11.53	59.55 ± 10.18	1.29	0.28
BMI (kg/m^2^) ^a^	24.06 ± 3.45	25.02 ± 3.45	23.64 ± 3.03	3.45	0.03
Fasting Blood Glucose (mmol/L) ^a^	6.61 ± 2.34	6.38 ± 2.56	6.34 ± 1.44	0.37	0.69
Systolic Blood Pressure(mmHg) ^a^	138.4 ± 23.39	140.86 ± 23.54	137.57 ± 26.20	0.39	0.68
Diastolic Blood Pressure (mmHg) ^a^	85.05 ± 12.31	87.68 ± 12.48	85.47 ± 12.83	0.99	0.37
Sex ^b^				0.15	0.94
Male	35 (42.20%)	33 (39.80%)	33 (39.80%)		
Female	48 (57.80%)	50 (60.20%)	50 (60.20%)		
Educational levela ^b^					
Primary school	55 (66.30%)	53 (64.60%)	63 (75.90%)	−0.08	0.21
Junior high school	24 (28.90%)	24 (29.30%)	16 (19.30%)		
Senior high school	4 (4.80%)	5 (6.10%)	4 (4.80%)		
Annual household income ^b^					
<1.2 w	30 (36.10%)	34 (41.00%)	26 (31.30%)	0.04	0.54
1.2–2 w	17 (20.50%)	24 (28.90%)	18 (21.70%)		
>2 w	36 (43.40%)	25 (30.10%)	39 (39.00%)		
Smoke ^b^				0.16	0.92
YES	25 (30.10%)	23 (27.70%)	23 (27.70%)		
NO	58 (69.90%)	60 (72.30%)	60 (72.30%)		
Alcohol ^b^				1.19	0.55
YES	26 (31.30%)	27 (32.50%)	21 (25.30%)		
NO	57 (68.70%)	56 (67.50%)	62 (74.70%)		
Colds(every year) ^b^					
0–4 times	82 (98.80%)	78 (94.00%)	76 (91.60%)	4.37	0.04
more than 4 times	1 (1.20%)	5 (6.00%)	7 (8.40%)		

^a^ continuous variables were expressed as mean ± standard deviation or median (P25, P75) and were compared between groups by analysis of variance (the Statistical magnitude is F). ^b^ categorical variables are expressed as numbers (percentages) and analyzed by chi-square test (the Statistical magnitude is χ^2^).

## Data Availability

The original contributions presented in the study are included in the article; further inquiries can be directed to the corresponding authors.

## Supplementary Materials

The following supporting information can be downloaded at: https://www.mdpi.com/article/10.3390/biom15050647/s1, Figure S1: Original Western blot images; Figure S2: Original Western blot images.
