# Supplementary figures and images for: Low Fluoride Regulates Macrophage Polarization Through Mitochondrial Autophagy Mediated by PINK1/Parkin Axis
Source: Biomolecules. 2025 Apr 30;15(5):647. doi: 10.3390/biom15050647 (PMC12109382; doi:10.3390/biom15050647)

Figure S1:Original Western blot images

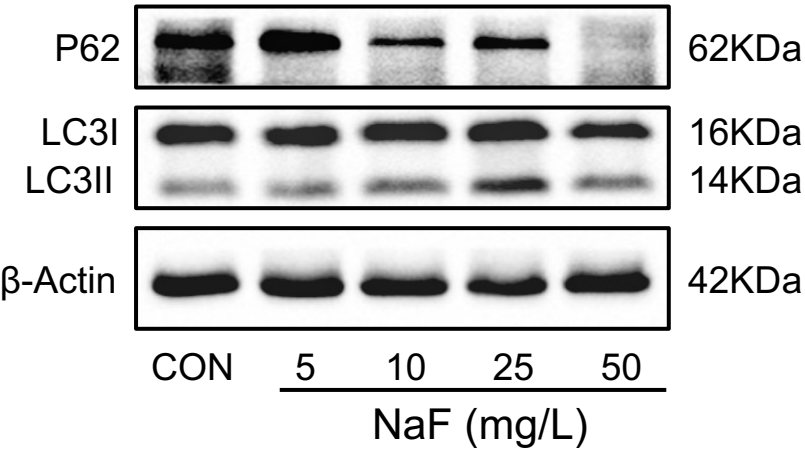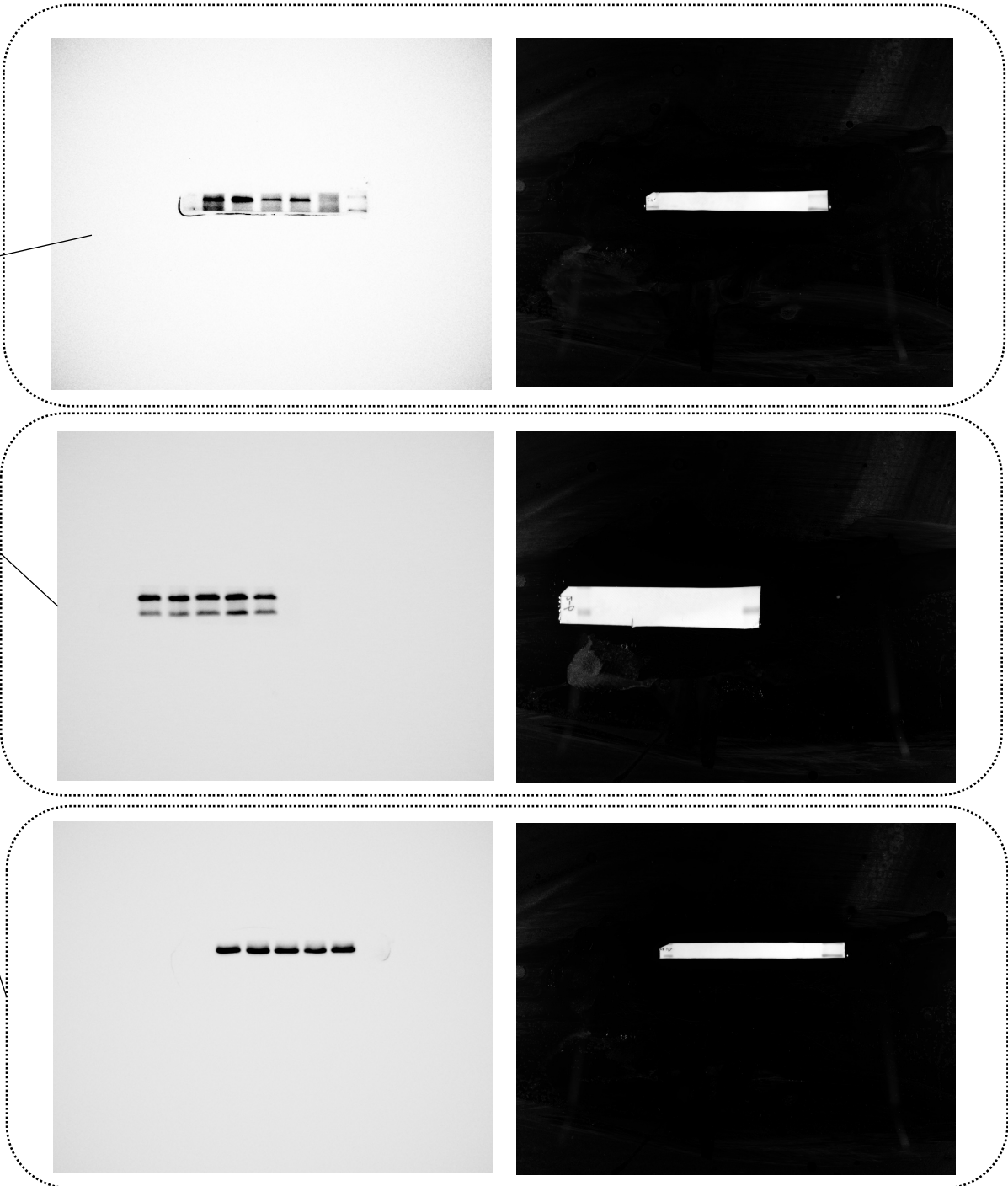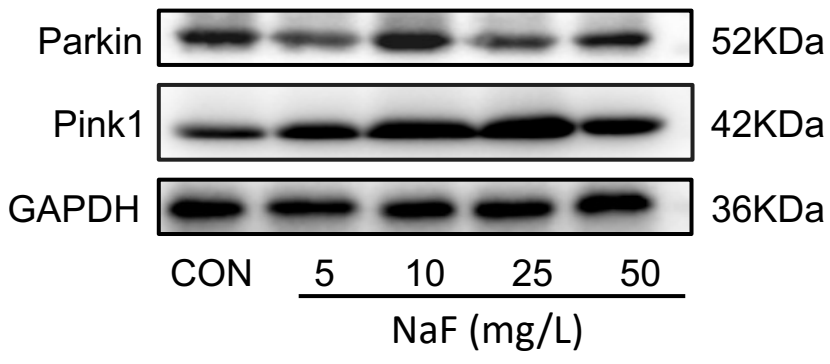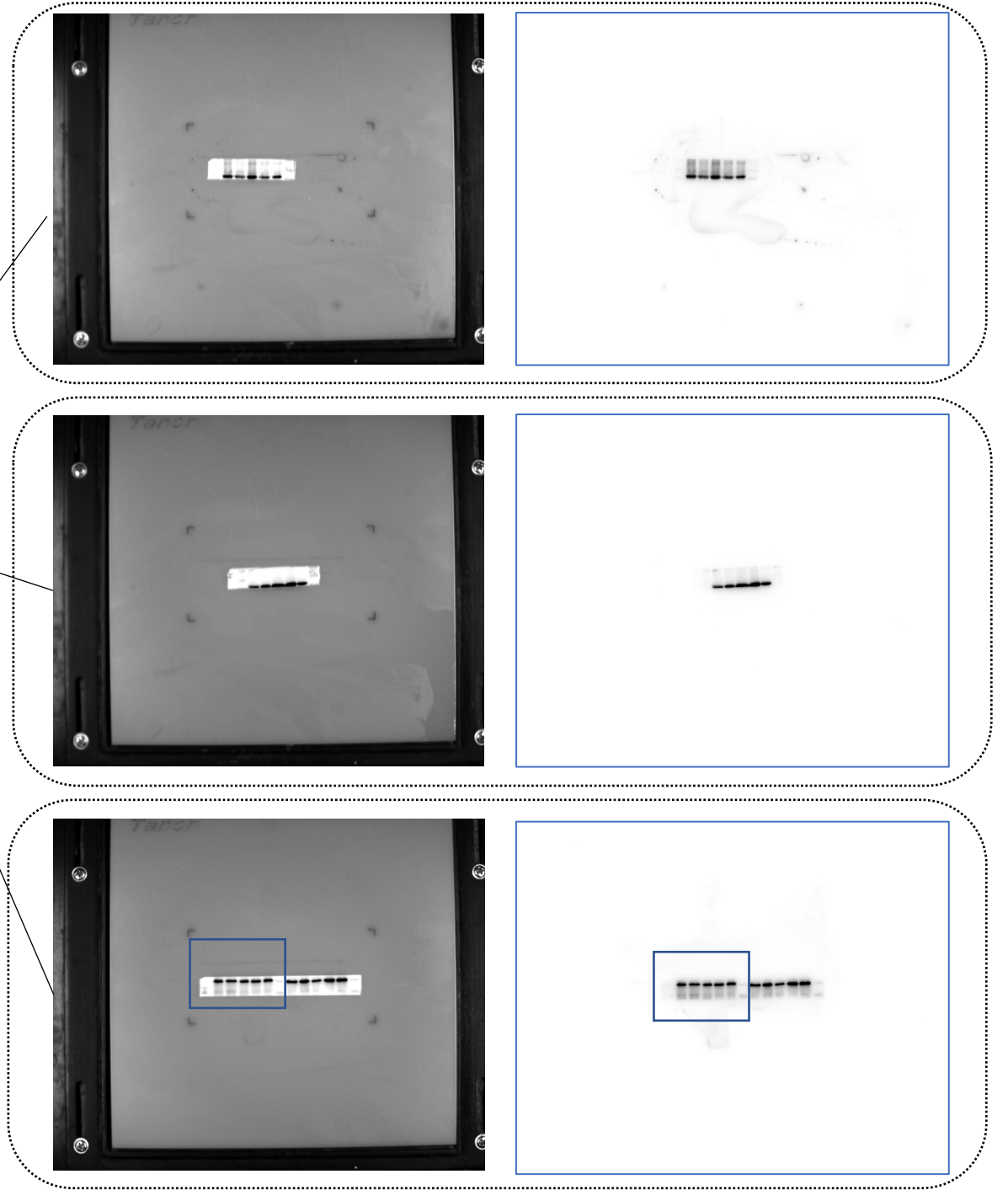

**Figure S2 : Original Western blot images**

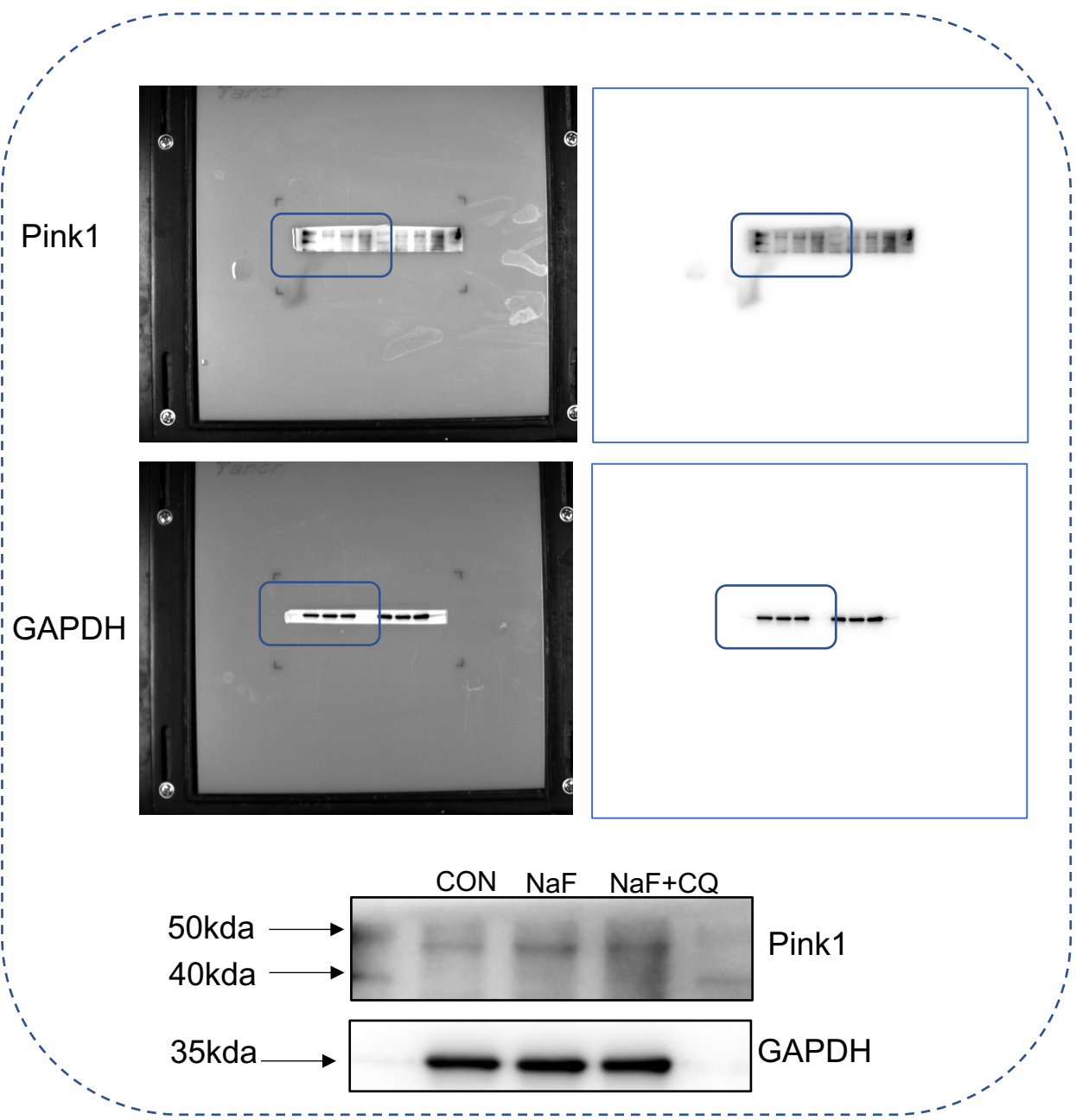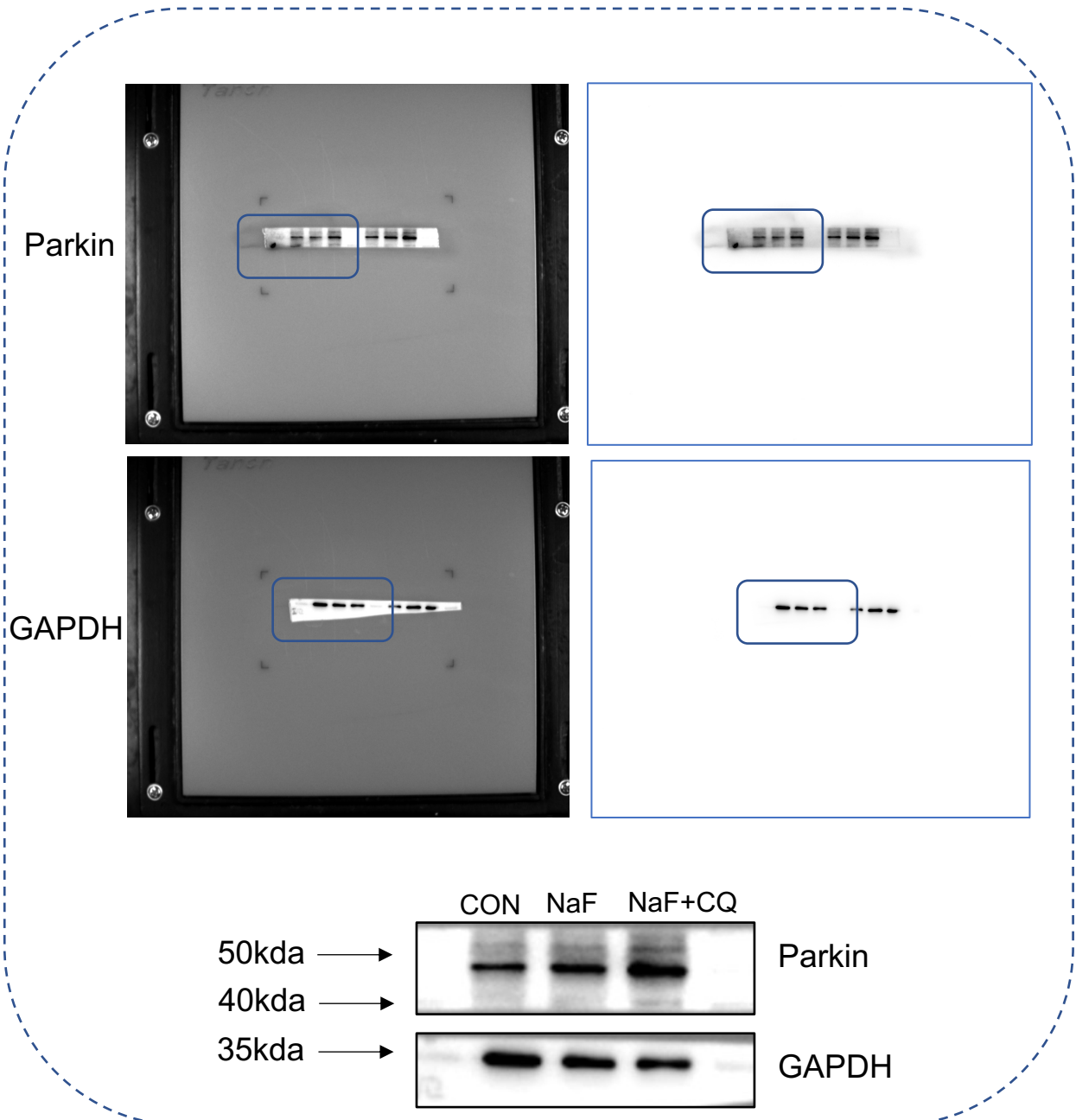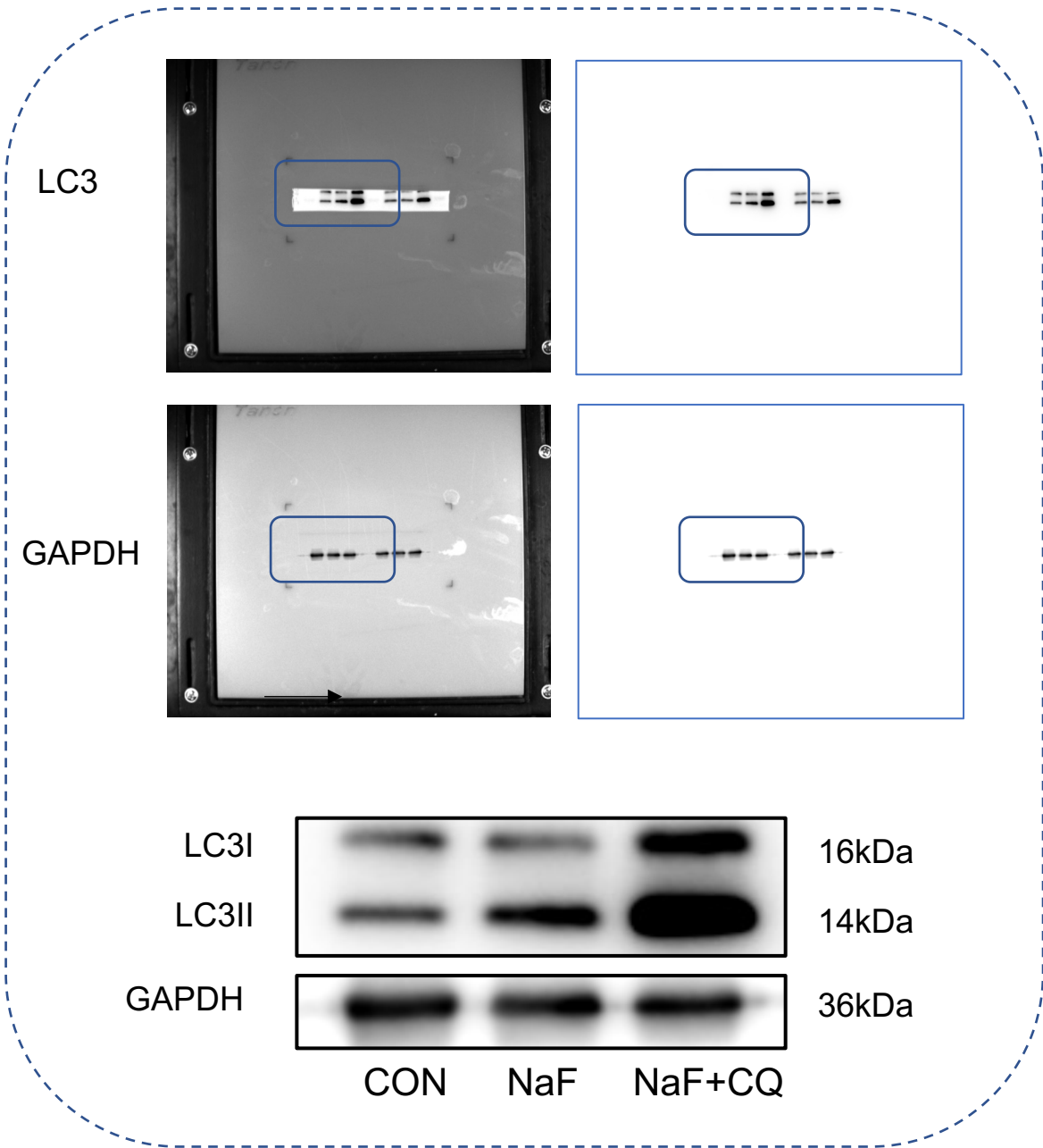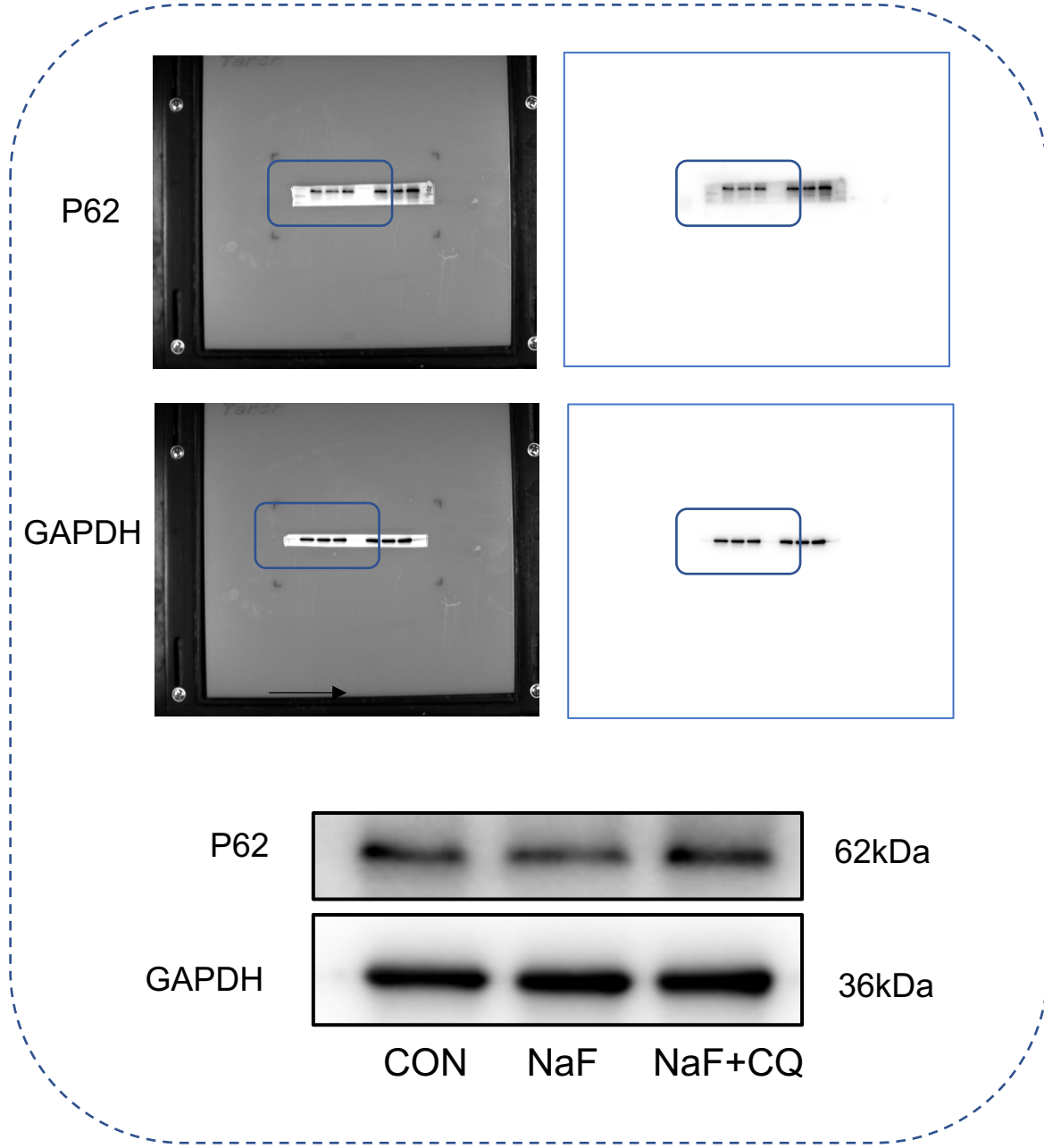

Supplement: Supplementary file 1 [file biomolecules-15-00647-s001.zip › biomolecules-3550348-supplementary.pdf]
